# Catabolic cytokine expression in degenerate and herniated human intervertebral discs: IL-1β and TNFα expression profile

**DOI:** 10.1186/ar2275

**Published:** 2007-08-09

**Authors:** Christine Lyn Le Maitre, Judith Alison Hoyland, Anthony J Freemont

**Affiliations:** 1 Tissue Injury and Repair Group, School of Medicine, Faculty of Medical and Human Sciences, The University of Manchester, Oxford Road, Manchester M13 9PT, UK

## Abstract

Low back pain is a common and debilitating disorder. Current evidence implicates intervertebral disc (IVD) degeneration and herniation as major causes, although the pathogenesis is poorly understood. While several cytokines have been implicated in the process of IVD degeneration and herniation, investigations have predominately focused on Interleukin 1 (IL-1) and tumor necrosis factor alpha (TNFα). However, to date no studies have investigated the expression of these cytokines simultaneously in IVD degeneration or herniation, or determined which may be the predominant cytokine associated with these disease states. Using quantitative real time PCR and immunohistochemistry we investigated gene and protein expression for IL-1β, TNFα and their receptors in non-degenerate, degenerate and herniated human IVDs. IL-1β gene expression was observed in a greater proportion of IVDs than TNFα (79% versus 59%). Degenerate and herniated IVDs displayed higher levels of both cytokines than non-degenerate IVDs, although in degenerate IVDs higher levels of IL-1β gene expression (1,300 copies/100 ng cDNA) were observed compared to those of TNFα (250 copies of TNFα/100 ng cDNA). Degenerate IVDs showed ten-fold higher IL-1 receptor gene expression compared to non-degenerate IVDs. In addition, 80% of degenerate IVD cells displayed IL-1 receptor immunopositivity compared to only 30% of cells in non-degenerate IVDs. However, no increase in TNF receptor I gene or protein expression was observed in degenerate or herniated IVDs compared to non-degenerate IVDs. We have demonstrated that although both cytokines are produced by human IVD cells, IL-1β is expressed at higher levels and in more IVDs, particularly in more degenerate IVDs (grades 4 to 12). Importantly, this study has highlighted an increase in gene and protein production for the IL-1 receptor type I but not the TNF receptor type I in degenerate IVDs. The data thus suggest that although both cytokines may be involved in the pathogenesis of IVD degeneration, IL-1 may have a more significant role than TNFα, and thus may be a better target for therapeutic intervention.

## Introduction

Intervertebral disc (IVD) degeneration and IVD herniation are major causes of low back pain (LBP) [1], which is a common, debilitating and economically important disorder [2,3]. However, none of the current treatments for LBP are directed at the altered cell and matrix biology underlying IVD degeneration or IVD herniation. Recent advances in therapeutics, particularly cell and tissue engineering, offer potential methods for inhibiting or reversing IVD degeneration, which has not previously been possible. However, the pathogenesis of IVD degeneration and IVD herniation is still not fully understood, and a greater understanding is necessary before such therapies can be fully developed for successful translation in the clinic.

The cells of the IVD behave abnormally during IVD degeneration, with decreased synthesis of the normal IVD matrix and increased production of degradative enzymes leading to a loss of the normal homeostatic metabolism in the IVD [4-7]. As a result there is destruction of the matrix with loss of hydration, resulting in spinal instability and a reduced ability to withstand load. Furthermore, IVD degeneration can also precede herniation of the IVD, which results in local nerve irritation, inflammation and further pain. In addition to the matrix degrading enzymes, these catabolic processes are thought to be mediated by a number of soluble mediators, including IL-1, tumour necrosis factor TNFα, IL-6, IL-8 and prostaglandin E_2 _[8-10].

Of these, the cytokines IL-1 and TNFα have been the focus of a number of studies investigating the pathogenesis of IVD degeneration, herniation and sciatic pain [11-20]. TNFα has been linked to IVD herniation and nerve irritation by a number of studies and the outcome of recent experiments using TNFα inhibitors has implicated this cytokine as an important mediator in LBP [15-20], whilst IL-1 has been shown to be directly involved in the decreased matrix synthesis and increased matrix degradation associated with IVD degeneration [12]. Importantly, elevated levels of IL-1 and TNFα have been found in aged and degenerative IVDs from both animal models and humans [12,21,22]. We have previously demonstrated the synthesis of IL-1α, IL-1β, IL-1 receptor type I (RI), IL-1β converting enzyme and IL-1Ra by the resident chondrocyte-like cells in human IVDs with significant increases in IL-1α, IL-1β, IL-1RI and IL-1β converting enzyme, but not IL-1Ra, during IVD degeneration [12]. Weiler and colleagues [21] found a positive correlation between TNFα and IVD degeneration, with approximately 80% of nucleus pulposus (NP) and 75% of annulus fibrosus (AF) cells staining positively for this cytokine. However, although Weiler and colleagues demonstrated an increase in TNFα immunopositivity in surgical samples compared to autopsy controls, the surgical samples were derived from a mixture of both herniated and degenerate IVDs and, thus, it was unclear from this study whether increased TNFα immunopositivity was observed in both disorders or just in herniation [21]. A recent study by Bachmeier and colleagues [22] also investigated the protein expression of TNFα, TNF receptors and the TNFα activating enzyme TACE in human IVD and demonstrated expression of all four molecules. Interestingly, although TNFα receptors were observed in autopsy samples, no results were presented for TNF receptor expression in surgical samples, thus raising the question as to whether TNFα is biologically active in such samples [22].

Thus, to date, it is not apparent whether both cytokines are involved in IVD degeneration or herniation and, if so, whether one has a predominant role in each disease state, an important question if future therapies are to be successful at targeting the processes involved in IVD degeneration and herniation.

Here, we use fully quantitative real time PCR and immunohistochemistry to investigate the gene and protein expression of IL-1β, TNFα and their receptors in non-degenerate, degenerate and herniated human IVDs to investigate whether both cytokines are expressed during IVD degeneration and herniation, and whether one may have a more predominant role.

## Materials and methods

### Tissue selection and grading of IVDs

Human IVD tissue was obtained either at surgery or post-mortem examination with informed consent of the patient or relatives. Local research ethics committee approval was given for this work by the following Local Research Ethics Committees: Salford and Trafford (Project number 01049), Bury and Rochdale (BRLREC 175(a) and (b)), Central Manchester (Ref No: C/01/008) and her Majesty's coroner (LMG/RJ/M6).

#### Post-mortem tissue

Previous studies have shown that IVD cells remain viable for at least 48 hours following death. In all, 8 IVDs were recovered from 6 patients within 18 hours of death (Table 1). They consisted of full thickness wedges of IVD of 120° of arc removed anteriorly, allowing well-orientated blocks of tissue to be cut for histological study. Patients with a history of sciatica or low back pain sufficient to warrant seeking medical opinion, were excluded from the study.

**Table 1:** Patient details and grades of tissues used for immunohistochemical analysis

Laboratory number	Source	Sex	Age (years)	MRI diagnosis	IVD level	Histological grade
1	PM	M	53	Not applicable	L4/5	1
2	PM	M	53	Not applicable	L5/S1	1
3	Surgical	M	44	Relatively normal	L4/5	1
4	Surgical	M	47	Relatively normal	L4/5	2
5	PM	M	75	Not applicable	L5/S1	3
6	Surgical	M	35	Mild degeneration	L5/S1	3
7	Surgical	M	48	Mild degeneration	L3/4	3
8	Surgical	F	64	Mild degeneration	L5/S1	3
9	Surgical	M	46	Normal	L5/S1	4
10	Surgical	M	21	Mild degeneration	L5/S1	4
11	Surgical	F	36	Mild degeneration	L5/S1	4
12	Surgical	M	25	Degenerate	L4/5	5
13	Surgical	F	32	Degenerate	L5/S1	5
14	Surgical	F	36	Degenerate	L4/5	5
15	Surgical	M	25	Degenerate	L4/5	5
16	Surgical	F	35	Degenerate	L4/5	6
17	Surgical	M	39	Degenerate	L4/5	6
18	PM	F	73	Not applicable	L5/S1	6
19	Surgical	M	25	Degenerate	L5/S1	6
20	Surgical	F	55	Degenerate	L5/S1	7
21	PM	F	Not known	Not applicable	L4/5	7
22	Surgical	F	58	Degenerate	L2/3	7
23	Surgical	M	34	Degenerate	L4/5	8
24	Surgical	F	24	Degenerate	L5/S1	8
25	Surgical	F	33	Severe degeneration	L5/S1	9
26	PM	F	73	Not applicable	L4/5	9
27	Surgical	M	68	Severe degeneration	L5/S1	10
28	PM	M	47	Not applicable	L5/S1	10
29	PM	M	47	Not applicable	L5/S1	11
30	Surgical	M	39	Severe degeneration	L4/5	12
31	Surgical	M	26	Herniated IVD	L5/S1	6
32	Surgical	F	43	Herniated IVD	L5/S1	7
33	Surgical	F	39	Herniated IVD	L4/5	7
34	Surgical	F	25	Herniated IVD	L5/S1	7
35	Surgical	M	35	Herniated IVD	L4/5	7
36	Surgical	M	44	Herniated IVD	L5/S1	9
37	Surgical	M	64	Herniated IVD	L5/S1	9
38	Surgical	M	28	Herniated IVD	L4/5	9
39	Surgical	F	45	Herniated IVD	L5/S1	10

IVD, intervertebral disc; F, female; M, male; PM, post-mortem.

#### Degenerate IVD tissue

Patients were selected on the basis of MRI diagnosed degeneration and progression to anterior resection either for spinal fusion or IVD replacement surgery for chronic low back pain. Patients experiencing classical sciatica were excluded from the study. Some patients underwent fusion at more than one level because of instability.

### Herniated IVD samples

Patients were selected on the basis of MRI diagnosed IVD herniation and progression to surgery for LBP for removal of the herniated material.

### General procedure for tissue specimens for immunohistochemical analysis

A block of tissue, incorporating AF and NP in continuity (or fragments of IVD for herniated samples), was fixed in 10% neutral buffered formalin and processed to paraffin wax. As some specimens contained bone, all the samples were decalcified in EDTA until radiologically decalcified. Sections were taken for haematoxylin and eosin staining to score the degree of morphological degeneration according to previously published criteria [6]. In brief, sections were scored for the presence of cell clusters, fissures, loss of demarcation and haematoxophilia (indicating reduced proteoglycan content): a score of 0 to 3 indicates a histologically normal (non-degenerate) IVD and a grade of 5 to 12 indicates evidence of degeneration. Tissue samples from 39 IVDs were selected for immunohistochemical analysis; these consisted of 8 non-degenerate IVDs (3 post-mortem samples and 5 surgical samples from patients where multiple disc levels were removed due to spinal instability), 22 degenerate IVDs (5 post-mortem samples and 17 surgical samples) and 9 herniated IVDs (all surgical) (Table 1).

### General procedure for tissue specimens for gene expression analysis

Tissue samples were divided into two and half the tissue incorporating AF and NP in continuity where present (or fragments of IVD for herniated samples) was taken for grading as described previously. Remaining tissue was separated into NP and AF tissue where both were present, finely minced and digested with 2 U/ml protease (Sigma, Poole, UK) in DMEM + F12 media for 30 minutes at 37°C and washed twice in DMEM + F12. NP cells were isolated in 2 mg/ml collagenase type 1 (Gibco, Paisley, UK) for 4 hours at 37°C. (Previous studies have shown 4 hour collagenase treatment does not affect gene expression in IVD cells (data not shown)). Immediately following cell extraction, RNA was extracted with Trizol^® ^reagent (Invitrogen, Paisley, UK)) and cDNA synthesized using Bioscript RNase H minus reverse transcriptase (Bioline Ltd, London, UK)) and random hexamers (Roche, East Sussex, UK)). RNA was extracted and cDNA synthesized from 64 lumbar IVD samples (NP and AF samples) for gene expression analysis (consisting of 24 non-degenerate (aged 37 to 61 years, mean age 51 years), 26 degenerate (aged 28 to 64 years, mean age 44.07 years) and 14 herniated (aged 20 to 51 years, mean age 29.15 years)).

### Gene expression for IL-1 and TNFα and their cytokines in human IVDs

Real time PCR was performed for genes encoding IL-1β, TNFα, IL-1 RI and TNF RI and the housekeeping gene 18s.

#### Primers and probe design

Primers and probes were designed using the Primer Express program (Applied Biosystems, Warrington, UK) within a single exon to allow detection of target genes in genomic DNA and cDNA samples. Total gene specificity was confirmed by BLAST searches (GenBank database sequences). Primers and probes were purchased from Applied Biosystems (Table 2).

**Table 2:** PCR primer and probe sequences and efficiencies

Target	Forward primer	Probe	Reverse primer	Efficiency (percent)
18s	PDAR	PDAR	PDAR	99.65
IL-1β	5' CGG CCA CAT TTG GTT CTA AGA 3'	5' ACC CTC TGT CAT TCG CTC CCA CA 3'	5' AGG GAA GCG GTT GCT CAT C 3'	90.5
TNF α	5' TGG TGG TCT TGT TGC TTA AAG TTC 3'	5' TCC CCT GCC CCA ATC CCT TTA TTA CCC G 3'	5' CGA ACA TCC AAC CTT CCC AAA C 3'	90.1
IL-1 RI	5' ATT TCT GGC TTC TAG TCT GGT GTT C 3'	5' ACT TGA TTT CAG GTG AAT AAC GGT CCC C 3'	5' AAC GTG CCA GTG TGG AGT GA 3'	98.5
TNF RI	5' CCT GGC CCC AAA CCC AAG 3'	5' TTC AGT CCC ACT CCA GGC TTC ACC C 3'	5' GTA TAG GTG GAG CTG GAG GTG 3'	93.8

RI, receptor type I; TNF, tumour necrosis factor. PDAR, Pre-developed assay reagents.

#### PCR amplification and quantification

PCR reactions were performed and monitored using the ABI Prism 7000 Sequence detection System (Applied Biosystems) as described previously [23]. For each gene, Taqman quantitative PCR was applied to 100 ng cDNA from each sample and genomic standard curve included on each real time plate. Copy number of each gene was determined by reference to the standard curve, generated from the genomic DNA standards. Copy numbers were then normalized to the real time expression of the housekeeping gene 18s as described previously [23]. Mann Whitney U tests were performed to analyse statistical differences between disease states for each gene investigated.

#### Production and localisation of IL-1β, TNFα and their receptors in human IVD

Immunohistochemistry was used to localise IL-1β, TNFα and their active receptors in 39 IVD samples (Table 1). The immunohistochemistry protocol followed was as previously published [12]. Briefly, 4 μm paraffin sections were dewaxed, rehydrated and endogenous peroxidase blocked using hydrogen peroxide. After washing in dH_2_O, sections were then treated with chymotrypsin enzyme antigen retrieval system (0.01% w/v chymotrypsin (Sigma), 20 minutes at 37°C) for IL-1β, TNFα and TNF RI. No enzyme retrieval was necessary for IL-1 RI. Following washing, non-specific binding sites were blocked at room temperature for 45 minutes with either: 20% w/v rabbit serum (Sigma) for TNFα, IL-1 RI and TNF RI; or 20% w/v donkey serum (Sigma) for IL-1β. Sections were incubated overnight at 4°C with mouse monoclonal primary antibodies against human TNFα (1:100 dilution; AbCam, Cambridge, UK), IL-1 RI (1:50 dilution; R&D Systems, Abingdon, UK)), TNF RI (1:10 dilution; R&D Systems) and goat polyclonal primary antibodies against human IL-1β (1:300 dilution; SantaCruz, Santa Cruz, CA, USA)). Negative controls in which mouse or goat IgGs (Dako, Cambridgeshire, UK) replaced the primary antibody (at an equal protein concentration) were used.

After washing, sections reacted with mouse monoclonal antibodies were incubated in biotinylated rabbit anti-mouse antiserum (1:400; Dako), and sections reacted with goat polyclonal primary antibodies were incubated in a 1:300 dilution of biotinylated donkey anti-goat antiserum (SantaCruz), all for 30 minutes at room temperature. Disclosure of secondary antibody binding was by the streptavidin-biotin complex (Dako) technique with 3,3'-diaminobenzidine tetrahydrochloride solution (Sigma). Sections were counterstained with Mayers Haematoxylin (Raymond A Lamb, East Sussex, UK)), dehydrated and mounted in XAM (BDH, Liverpool, UK)).

### Image and statistical analysis

All slides were visualised using a Leica RMDB research microscope and images captured using a digital camera and Bioquant Nova image analysis system. Each section was divided into the NP, inner AF (IAF) and outer AF (OAF) where present, and analysed separately. Within each area 200 cells were counted and the number of immunopositive cells (brown staining) expressed as a proportion of this. Data were non-parametric and hence Mann Whitney U tests were performed to compare the numbers of immunopositive cells in degenerate and herniated groups to non-degenerate IVDs (scores 0 to 3) for each area of the IVD. In addition, Wilcoxon paired sample tests were used to compare proportions of immunopositive cells in the different areas of the IVDs. This analysis was performed using all IVD sections regardless of disease state.

## Results

### Gene expression of IL-1 and TNF and their receptors in non-degenerate human IVDs

**Figure 1:**
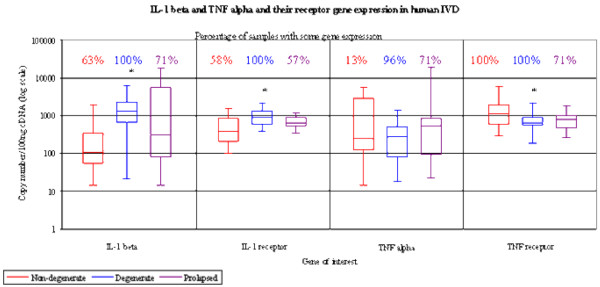
Absolute gene expression of IL-1β, tumour necrosis factor (TNF)α and their receptors in human intervertebral discs (IVDs). The percentage of disc samples displaying gene expression for the target genes and the copy number/100 ng cDNA expressed within positive samples are given and data is represented as a box and whisker plot. (* = P < 0.05).

IL-1β was expressed in more non-degenerate IVDs than those expressing TNFα (63% versus 13%). TNFα was expressed only in IVDs expressing IL-1β. By comparison, only 58% of non-degenerate samples displayed IL-1 RI gene expression compared to 100% of samples displaying TNF RI gene expression. In addition, samples where gene expression was seen for the receptors demonstrated higher copy numbers for TNF RI (1,087 copies/100 ng cDNA) than IL-1 RI (386 copies/100 ng cDNA) (Figure 1).

### Gene expression of IL-1 and TNF and their receptors in degenerate human IVDs

The proportion of IVD cells expressing IL-1β and TNFα genes was greater in degenerate (100% and 96%, respectively) than non-degenerate IVDs (63% and 13%, respectively). IL-1β gene copy number was greater in degenerate than non-degenerate IVDs (*P *< 0.05; Figure 1, Table 3), whereas there was no difference in TNFα copy number between non-degenerate and degenerate IVDs (Figure 1). In degenerate IVDs, the mean copy number was greater for IL-1β than TNFα (median of 1,298 copies of IL-1β gene/100 ng cDNA, and 277 copies of TNF alpha/100 ng cDNA; Figure 1).

**Table 3:** Summary of gene and protein expression differences seen compared to non-degenerate discs

Target	Gene expression	Protein expression
**IL-1β**		
Degenerate discs	Proportion of samples ↑ and level ↑ (*P *< 0.05)	↑ in NP and IAF (*P *< 0.05)
Herniated discs	Proportion of samples ↑ (*P *< 0.05)	↑ in NP and IAF (*P *< 0.05)
**IL-1 receptor**		
Degenerate discs	Proportion of samples ↑ and level ↑ (*P *< 0.05)	↑ in NP (*P *< 0.05)
Herniated discs	Proportion of samples NC, level ↑ (*P *> 0.05)	↑ in NP (*P *< 0.05)
**TNFα**		
Degenerate discs	Proportion of samples ↑ (*P *< 0.05), level NC	↑ in NP and IAF (*P *< 0.05)
Herniated discs	Proportion of samples ↑ (*P *< 0.05), level NC	↑ in NP (*P *< 0.05)
**TNF receptor**		
Degenerate discs	Proportion of samples NC, level ↓ (*P *< 0.05)	↓ in NP and IAF (*P *> 0.05)
Herniated discs	Proportion of samples ↓ (*P *< 0.05), level NC	↓ in NP (*P *> 0.05)

Up and down arrows indicate increase and decrease, respectively. IAF, inner annulus fibrosus; NC, no change; NP, nucleus pulposus.

All degenerate IVDs expressed the genes for both cytokine receptors. This represented an increase over non-degenerate samples in the number of cases expressing the IL-1 RI gene (100% compared to 58%; Figure 1, Table 3). Degenerate IVDs also demonstrated significantly higher copy numbers for IL-1 RI than receptor positive non-degenerate IVDs (906 copies/100 ng cDNA in degenerate IVDs versus 386 copies/100 ng cDNA in non-degenerate IVDs; *P *< 0.05; Figure 1, Table 3). As for the non-degenerate IVDs, TNF RI was seen in all degenerate IVDs (Figure 1). However, degenerate IVDs showed significantly less copy numbers for TNF RI than seen in non-degenerate IVDs (651 copies/100 ng cDNA in degenerate IVDs versus 1,087 copies/100 ng cDNA in non-degenerate IVDs; *P *< 0.05; Figure 1, Table 3).

### Gene expression of IL-1 and TNF and their receptors in herniated human IVDs

IL-1β gene expression was observed in a greater number of herniated IVDs (71%) than non-degenerate IVDs (63%). The level of gene expression was also higher in herniated IVDs than non-degenerate IVDs, although this did not achieve statistical significance (*P *> 0.05; Figure 1, Table 3). Similarly, TNFα was also seen in a greater proportion of herniated IVDs (71%) than non-degenerate IVDs (13%). TNFα was seen in some herniated samples where IL-1β was not expressed, although the majority of samples expressing TNFα also expressed IL-1β. In addition, the level of TNFα gene expression was higher in herniated IVDs than that seen in non-degenerate and degenerate IVDs, although this did not achieve statistical significance (Figure 1, Table 3). No significant difference was seen between the level of gene expression for IL-1β and TNFα in herniated IVDs (301 copies/100 ng cDNA for IL-1β, and 520 copies/100 ng cDNA for TNFα; Figure 1).

IL-1 RI was seen in a similar proportion of herniated and non-degenerate IVDs but in fewer IVDs than in degenerate IVDs. A non-significant increase in levels of IL-1 RI was also seen in herniated IVDs compared to non-degenerate IVDs but at lower levels than in degenerate IVDs (Figure 1, Table 3). Expression of TNF RI was seen in a lower proportion of herniated IVDs, with only 71% of samples displaying expression compared to all non-degenerate and degenerate IVDs. The level of TNF RI expression was also lower in herniated IVDs than in non-degenerate IVDs, although this did not reach statistical significance (Figure 1, Table 3).

### Protein production and localisation of IL-1 and TNF and their receptors in human IVDs

**Figure 2:**
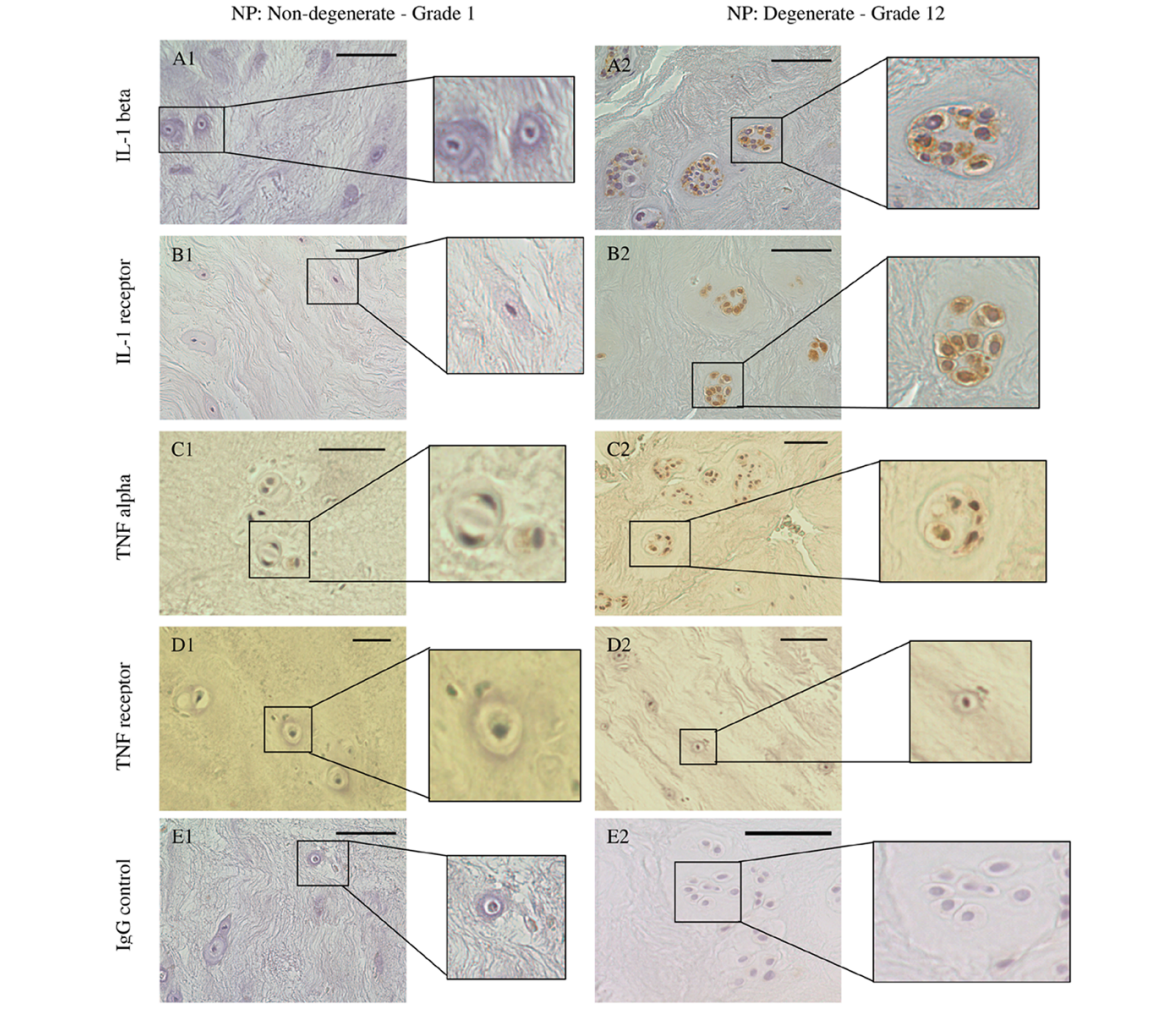
Photomicrographs illustrating immunohistochemistry staining for IL-1β, tumour necrosis factor (TNF)α and their receptors in human intervertebral discs. Results for non-degenerate discs (grade 1) are shown in A1 to E1 and result for degenerate discs (grade 12) are shown in A2 to E2: IL-1β (A); IL-1RI (B); TNFα (C); TNF RI (D); IgG controls (E) were all negative. Immunopositivity shows as brown staining. Bars = 570 μm.

**Figure 3:**
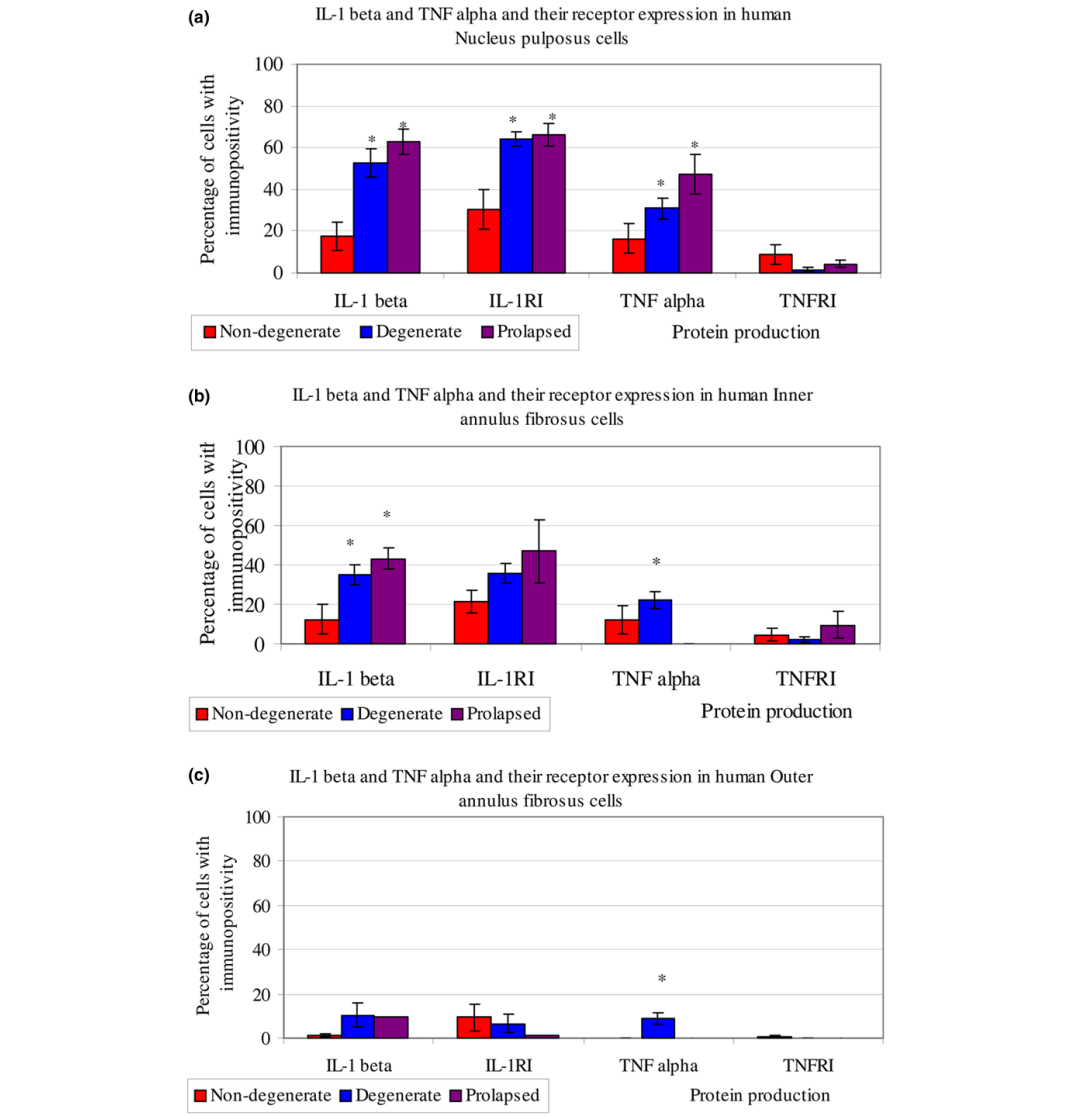
Number of cells displaying immunopositivity for IL-1β, tumour necrosis factor (TNF)α and their receptors in human human intervertebral discs. The percentage of cells with immunopositivity is given for IL-1β, IL-1RI, TNFα and TNF RI in the **(a) **nucleus pulposus, **(b) **inner annulus fibrosus and **(c) **outer annulus fibrosus of non-degenerate, degenerate and herniated discs (*n *= 39). Data are presented as means ± 2 standard error (as a representative of 95% confidence interval). *P < 0.05.

Immunoreactivity for the four molecules (IL-1β, TNFα, IL-1 RI and TNF RI) was observed in non-degenerate, degenerate and herniated IVDs. The immunostaining was generally restricted to the cytoplasm of native IVD cells (Figure 2). IgG controls were always negative (Figure 2). No immunopositivity was observed in the matrix of the IVD or in blood vessels. Staining was particularly prominent in the cytoplasm of the chondrocyte-like cells of the NP and IAF, with significantly lower numbers of cells in the OAF showing immunopositivity for all four targets investigated (*P *< 0.05). IL-1β and its receptor showed significantly more immunopositive cells in the NP than the IAF and the OAF (*P *< 0.05; Figure 3).

In non-degenerate IVDs, a similar proportion of IVD cells were immunopositive for IL-1β and TNFα, with approximately 20% of cells in the NP and 10% in the IAF being immunopositive (Figure 3, Table 3). However, a greater proportion of cells (30% of cells in the NP and 20% of cells in the IAF) were immunopositive for IL-1 RI than TNF RI (10% of cells in the NP and 5% of cells in the IAF). The percentage of cells immunopositivite for IL-1β and TNFα was significantly increased in the NP and IAF of degenerate IVDs compared to non-degenerate IVDs (*P *< 0.05; Figure 3, Table 3). However, this increase was greater for IL-1β than TNFα, with approximately 50% of cells in degenerate IVDs showing IL-1β immunopositivity but only 30% TNFα immunopositive cells. The percentage of cells immunopositivite for IL-1 RI was higher in degenerate than non-degenerate IVDs, although this only reached significance in the NP (*P *< 0.05; Figure 3, Table 3). However, no such increase was seen for TNF RI, where the number of immunopositive cells was low (3%); numbers of TNF RI immunopositive cells actually decreased in degenerate discs compared to non-degenerate discs, although this did not reach significance (Figure 3, Table 3).

When compared to non-degenerate IVDs, herniated IVDs also showed significantly higher numbers of cells immunopositive for IL-1β, TNFα and IL-1 RI but not TNF RI (Figure 3, Table 3). The proportion of cells immunopositive for IL-1β and IL-1 RI was similar in herniated and degenerate IVDs, but a greater proportion of TNFα immunopositivite cells was seen in herniated than degenerate IVDs.

## Discussion

The pathogenesis of IVD degeneration is still poorly understood, and a greater understanding is required prior to the development of successful therapeutic approaches to inhibit or delay IVD degeneration and herniation and thus treat LBP. A number of cytokines have been implicated in the pathogenesis of IVD degeneration and herniation, with particular attention being paid to IL-1 and TNFα [11-20]. To our knowledge, this is the first study to simultaneously investigate the gene expression and protein production of IL-1 and TNFα and their receptors in non-degenerate, degenerate and herniated human IVDs.

This study demonstrated that IL-1 and TNFα are expressed along with their receptors in the human IVD. Both cytokines were present in non-degenerate IVDs at low levels, with similar numbers of immunopositive cells seen, although the gene expression analysis suggested that IL-1β was more highly expressed than TNFα in non-degenerate IVDs. TNF RI gene expression was observed in all non-degenerate samples; however, immunohistochemistry showed only a small number of cells with TNF RI protein, suggesting that the gene was not translated to protein. An alternative explanation may be that real time PCR is more sensitive than immunohistochemisty and, thus, may have identified gene expression where protein levels could not be detected. IL-1 RI protein, however, was produced by a greater number of cells than TNF RI, suggesting that non-degenerate IVD cells were more responsive to the local levels of IL-1 than TNFα. This suggests that IL-1 is important in the normal homeostasis of the IVD where IL-1 is controlled by the natural antagonist IL-1Ra, which we have previously shown to be synthesized by endogenous IVD cells [12].

During IVD degeneration and IVD herniation, an increase in the protein production for both cytokines was observed, agreeing with the two earlier studies on protein production of these cytokines in degenerate human IVDs [12,21]. However, the number of IL-1β immunopositive cells was higher than the number of TNFα immunopositive cells in both degenerate and herniated samples. In addition, gene expression for IL-1β but not TNFα was significantly increased in degenerate compared to non-degenerate IVDs.

This study also showed an increase in both IL-1RI gene expression and protein production in degenerate and herniated IVDs compared to non-degenerate IVDs, a finding that agrees with our earlier study in which we demonstrated increased immunopositivity for IL-1RI in degenerate compared to non-degenerate IVDs [12].

The biological activity of TNF is mediated through two distinct but structurally homologous TNF receptors, type I (p60 or p55) and type II (p80 or p75). Although TNF binds to each with high affinity, TNF RI is more ubiquitously expressed and it is generally believed that TNF RI is responsible for the majority of biological actions of TNF while TNF RII may function to potentate the effects of TNF RI. We have previously shown that TNF RII is not expressed by IVD cells either in normal or degenerate IVDs [24]. The current study also shows the expression and production of TNF RI within human intervertebral IVDs, which together with the recent study by Bachmeier and colleagues [22] suggests that human IVD cells are capable of responding to TNFα *in vivo*. However, no increase in TNF RI synthesis was seen during IVD degeneration or herniation. In fact, a decrease in TNF RI gene and protein expression was seen in degenerate and herniated IVDs compared to non-degenerate IVDs, suggesting that the biological activity of TNFα is reduced during degeneration and herniation due to these IVDs having reduced responsiveness to TNFα.

We have previously demonstrated that in human IVD cells, IL-1 treatment results in decreased matrix production and increased production of the degradation enzymes (matrix metalloproteinases and ADAMTs (a disintegrin and metalloprotease with thrombospondin motifs)) [12], features characteristic of IVD degeneration [5,7]. Similar responses have been observed in animal IVD cells following treatment with TNFα [25], although these responses have not been shown to date in human IVDs. Here we demonstrate the expression and localisation of the TNF RI to the chondrocyte-like cells of the human intervertebral IVDs, and although only expressed in a low percentage of IVD cells, this suggests that the human IVDs are capable of responding to TNFα, which could result in decreased matrix synthesis and increased matrix degradation in a similar manner to that seen with IL-1. However, as our data demonstrated low expression of TNF RI in human IVD samples, this suggests that these effects would be limited.

Although our study indicates that TNFα may have limited effects on IVD cells during IVD degeneration, during IVD herniation the TNFα produced by IVD cells in the herniated IVDs may have additional detrimental effects. During IVD herniation, IVD tissue comes into contact with the nerve root and inflammatory cells. TNFα has been shown to result in the sensitisation of nerve roots and stimulation of nerve growth factor [15,26,27]. As such, the TNFα generated by the IVD cells in herniated IVD tissue could have a detrimental effect on the local nerves, resulting in the generation of LBP. Indeed, the use of TNF blocking antibodies has shown some promise in a small clinical trial [28,29], although two more recent placebo controlled trials suggest that this treatment may only be of use in a small subset of sciatica patients (that is, those with L4/5 or L3/4 herniation with modic changes) [30,31].

Our data suggest that TNFα, in addition to IL-1, may have a role in the pathogenesis of IVD degeneration. However, we have shown that IL-1 is expressed and produced at higher levels than TNFα, suggesting IL-1 may be more predominant in the processes of IVD degeneration. In addition, this study has highlighted that IL-1 RI expression by native IVD cells is upregulated during IVD degeneration and herniation, suggesting that there is increased responsiveness to IL-1 during these disease states. In contrast, TNF RI was only produced by a small proportion of IVD cells in non-degenerate IVDs and its expression and production were decreased during IVD degeneration and herniation, suggesting that the responsiveness of IVD cells to TNFα in degenerate human IVDs may only be at low levels.

The data presented in the current study together with our previous findings [12] suggest that IL-1 would be a viable target for the inhibition of disc degeneration. Indeed, IL-1Ra therapies such as Anakinra are already in clinical use for limiting cartilage degradation in rheumatoid arthritis and osteoarthritis and, as such, its pharmacology and side effects are increasingly well understood [32]. We have previously demonstrated successful transfer of IL-1Ra to IVD tissue *in vitro *using gene therapy and, thus, delivery to the IVD is a viable option [33]. The inhibition of IL-1 driven processes, leading to disc degeneration, could be important in two therapeutic strategies; first, inhibition of disc degeneration at an early stage (such as degeneration induced at adjacent levels following spinal fusion or disc replacement); and second, defining the optimal tissue niche for regenerating the end stage degenerate IVD.

## Conclusion

Our data show that both IL-1 and its receptor are significantly upregulated in IVD degeneration and are, therefore, more likely to be major mediators in the processes of IVD degeneration. By contrast, whilst TNFα expression is upregulated in degeneration, gene and protein expression of the predominant TNF receptor (TNF R1) is, if anything, reduced, with few cells showing demonstrable protein production. The implication, therefore, is that overall biological activity of TNFα within the degenerate IVD is reduced. The herniated IVD is, however, rather different. Although TNF RI expression is low, TNFα gene and protein expression are higher overall than in the non-degenerate IVD and whilst the biological activity of TNFα within the IVD tissue will still be restricted by the low receptor expression our results would support the data from others implicating a potential paracrine effect of TNF produced by IVD cells in inducing sciatica. To conclude, the results from this study suggest that IL-1 rather than TNFα would be a better target for therapeutic approaches to inhibit IVD degeneration and associated LBP.

## Competing interests

The authors declare that they have no competing interests.

## Authors' contributions

CLM helped conceive the study, participated in its design, performed all the laboratory work and analysis and drafted the manuscript. AJF helped to secure funding, participated in interpretation of data and contributed to the preparation of the final manuscript. JAH conceived the study, secured funding, contributed to its design and co-ordination, and participated in interpretation of data and co-wrote the manuscript. All authors read and approved the final manuscript.
